# Surface Lipids as Multifunctional Mediators of Skin Responses to Environmental Stimuli

**DOI:** 10.1155/2010/321494

**Published:** 2010-10-20

**Authors:** Chiara De Luca, Giuseppe Valacchi

**Affiliations:** ^1^Laboratory of Tissue Engineering and Skin Pathophysiology, Istituto Dermopatico dell'Immacolata (IDI IRCCS), Via Monti di Creta 104, 00167 Rome, Italy; ^2^Department of Food and Nutrition, Research Institute of Human Ecology, Kyung Hee University, Seoul 130-701, Republic of Korea; ^3^Department of Biomedical Sciences, University of Siena, 53100 Siena, Italy

## Abstract

Skin surface lipid (SSL) film is a mixture of sebum and keratinocyte membrane lipids, protecting skin from environment. Its composition is unique for the high percentage of long chain fatty acids, and of the polyterpenoid squalene, absent in other human tissues, and in non-human Primates sebum. Here, the still incomplete body of information on SSL as mediators of external chemical, physical, and microbial signals and stressors is revised, focusing on the central event of the continuous oxidative modification induced by the metabolic activity of residential and pathological microbial flora, natural or iatrogenic UV irradiation, exposure to chemicals and cosmetics. Once alpha-tocopherol and ubiquinol-10 antioxidant defences of SSL are overcome, oxidation of squalene and cholesterol gives rise to reactive by-products penetrating deeper into skin layers, to mediate local defensive inflammatory, photo-protective, immune reactions or, at higher concentrations, inducing local but also systemic immune depression, ultimately implicating skin cancerogenesis. Qualitative modifications of SSL represent a pathogenetic sign of diagnostic value in dermatological disorders involving altered sebum production, like pytiriasis versicolor, acne, atopic or seborrheic dermatitis, as well as photo-aging. Achievements of nutriceutical interventions aimed at restoring normal SSL composition and homeostasis are discussed, as feasible therapeutic goals and major means of photo-protection.

## 1. Sources and Composition of Skin Surface Lipids

A continuous hydrolipidic film, representing the actual interface between the epidermal viable layers and outer environment, covers human skin. Skin surface lipids (SSLs) are a mixture of sebaceous and epidermal lipids, displaying a very peculiar composition as compared to lipid fractions of serum or internal tissues. This peculiarity is originated by the unique contribution of sebum secreted from the sebaceous glands, unevenly distributed in all areas of the body with the exception of the palms and foot soles, and becoming extremely specialized in local districts, like the eye, where the meibomian glands exert highly efficient protective functions [1].

SSL composition in the skin areas with the highest concentration of sebaceous glands (forehead, upper chest, and dorsum) mainly reflects sebaceous secretion, flowing from those sites also to areas with lower concentration, where the contribution of cellular lipid components, rich in oleic and linoleic acid, becomes more relevant [2, 3]. The keratinocyte membrane lipid contribution and the continuous metabolic action of resident microbial flora hosted at skin surface in healthy conditions are key determinants of the uniqueness of this complex mixture. Major lipid components in human sebum include squalene (SQ—(2,6,10,15,19,23,-hexamethyl-2,6,10,14,18,22-tetracosahexaene), wax esters, and triglycerides. As a whole, the SSL fatty acids fraction is relatively poor in polyunsaturated fatty acids (PUFAs). Typically, sebum is rich in long-chain fatty acids (up to 26 carbon atoms), linear or branched, mainly saturated or monounsaturated [4, 5]. These are partly present in the free form, secondary to the microbial and epithelial lipase activity on sebum triglycerides, and are responsible for antimycotic and antibacterial properties of the skin [5–7]. For the most part, these specialized fatty acids are esterified with cholesterol, or with fatty alcohols, to form the fraction of wax mono- and diesters, crucial for skin insulation [8–10], available uniquely on the skin and hair shaft. This fraction has been extensively investigated by analytical lipidomic approach in recent years, allowing, for example, the identification of more than 160 different wax esters ranging from 24 to 42 total carbons, and 73 species of ceramides, in the human proximal hair [11, 12].

A most peculiar component of SSL is SQ [2], a key biosynthetic precursor of cholesterol. In humans, about 60 percent of dietary SQ is absorbed, transported in serum by very low-density lipoproteins, and distributed ubiquitously in human tissues, with the greatest accumulation in the skin through sebocyte concentration [13]. SQ levels, being negligible in other organs, normally range about 12% of total SSL in adult life and can reach up to 20% [14, 15]. In the liver and in other tissues, this linear 30-carbon triterpenoid compound is entirely metabolised to SQ 2,3-epoxide to be subsequently converted to lanosterol. SQ overproduction in sebocytes may be due to altered expression and activity of two key oxygen-regulated enzymes involved in SQ metabolism, squalene synthase, and squalene oxidocyclase, in response to the anaerobic environment occurring locally inside the sebaceous gland [16]. This biochemical peculiarity bears important biological implications, in that the peroxidable SQ molecule has been extensively proven to be a key mediator of skin reactions to environmental stressors [17].

In defence towards oxidative events occurring on the skin, vitamin E of nutritional origin, actively secreted from sebaceous glands and probably cosecreted with SQ [18] and coenzyme Q_10_ of endogenous origin and partly co-synthesized with SQ by the sebaceous gland [19], provide necessary antioxidant protection to the skin lipid film.

The cellular-derived component of skin surface lipids consists of phospholipids derived from the plasma membrane of corneocytes, also contributing as the unique component of ceramides characterizing cell envelope [7, 20]. Lipids from keratinizing cells account for the rather limited contribution of polyunsaturated fatty acids, namely linoleic acid [21], available in the surface hydrophobic film. More deeply into the cornified envelope, ceramides and proteins concur to form the unique intercellular matrix determining most of the functions of the skin barrier [22].

The overall composition of SSL differs therefore sensibly from lipids of the viable skin epithelial layers, and from systemic lipids, both in the relative percentage and type of lipid fractions, and in the relative amount of the PUFA oxidable fraction [16]. Following the rather extensive studies on the composition and role of SSL started by the pioneer observations of Nicolaides [10], the research on epidermal surface lipids and their prevalent sebaceous component has been thereafter almost entirely dropped, in favour of the studies on lipids of keratinocyte origin, thus unravelling the essential functions of ceramides in cell signalling and skin disease [23, 24]. Nevertheless, the role of SSL in mediating the biologic effects of UV irradiation and other environmental stimuli/stressors has been at least partly identified [25], and, most importantly, alterations in sebum secretion have been recognised as a common feature of several inflammatory chronic skin diseases, finding their main localization in cutaneous areas where sebaceous glands are more concentrated. In addition, SSL chemical composition can be severely altered in different skin diseases. This is the case of acne [26–28], atopic [29] and seborrheic dermatitis [30], pityriasis versicolor [31, 32], and androgenic alopecia [33]. Sebum must in fact also be regarded as vehicle transporting and transmitting several endogenous and exogenous molecules to the skin, including potential regulatory factors of hair follicles [34].

All the above-mentioned dermatoses are so far lacking satisfactory aetiopathogenic elucidation and consequently established and effective therapies. Interestingly, all of them benefit from therapeutic UV irradiation protocols [35].

## 2. Effects on SSL of Natural UV Irradiation and Phototherapy

Among the different categories of environmental stressors affecting the skin, UV irradiation is the field of most intense research targeting the increased risk of tumor development connected with overexposure and the effects on cutaneous pigmentation of medical/aesthetic concern. Based on the capability of UV rays to penetrate at different depths in the epidermal and dermal layers [36], most of the studies have been so far focused on the photoreceptors localised in the skin viable compartments beneath corneum, such as DNA, urocanic acid [37], and endogenous [38] or bacterial porphyrins [39]. These molecules are able to selectively absorb in the UV wavelenght range and thus catalyse oxidative cytostatic, cytotoxic, or imunomodulating photoreactions, widely employed for medical applications in skin phototherapy.

Indeed, the very first target of UV and other environmental radiations during natural, professional, or therapeutic exposure is represented by skin surface lipids [40]. Nevertheless, their possible role as mediators of cutaneous and systemic biological effects has been so far not adequately highlighted. The UV-R absorbance spectrum for SSL has been assessed spectrometrically, showing significant absorption, with a maximum at 215 nm. The hydrolipid layer present on forehead skin in healthy conditions is likely to reduce transmission at 300 nm by about 10% [41]. Apart from representing a first line of defence by direct UV absorption, SSL constitutes a suitable target and scavenger for all reactive species generated at skin level by different molecular mechanisms, in the course of UV irradiation. This indirect photo-oxidation is definitely the most relevant biological effect of UV on the SSL mantel and is the main mechanism operating in ultraviolet A- (UVA-) and visible light-induced photodynamic stress induced on skin for therapeutical purposes [42]. Due to the scavenging action of SSL under atmospheric oxidative chemical stress, photoirradiation, or microbial oxidative metabolism, the hydrolipid superficial mantel becomes a relevant source of peroxidated intermediates on the skin, certain molecules of the resulting composition being clearly cytotoxic, irritant, or immunogenic [34].

**Figure 1:**
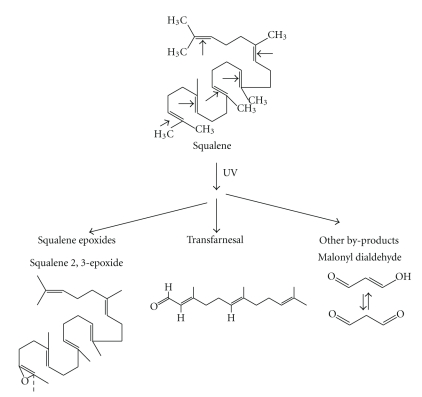
Chemical structures of squalene and of its main photo-oxidation labile or stable by-products, generated *in vitro* under UV irradiation.

Among cutaneous SSL components, squalene is a most intriguing component. It represents indeed the most abundant peroxidable fraction in SSL. It was previously demonstrated that when human sebum is subjected to high-dosage UVB irradiation *in vitro*, SQ is markedly degraded as compared to cholesterol, sebum triglycerides, or mono-/diunsaturated free fatty acids (FFAs) [43, 44]. UVR, alone or in combination with photosensitizers or other physicochemical stimuli including microbial peroxidizing metabolism, induces oxidative degradation of SQ with the generation of a wide range of by-products of varying polarity and reactivity, that have been clearly characterised by ion mass spectrometry. These include SQ monohydroperoxide [43], different isomers of squalene epoxide, and shorter chain reactive aldehydes [45], in particular formaldehyde and malonyl dialdehyde (MDA) [46–48] ] (Figure 1). More recent studies have demonstrated that SQ is oxidized at a much higher rate in physiological conditions by UVA either than UVB, and that probably most previous results obtained with experimental UVB irradiation must be ascribed to the effects of minimal doses of UVA, always contaminating UVB emission sources [49].

Following the initial studies concentrated on SQ oxidation dynamic, conclusive works have indicated that the very first molecules undergoing oxidation *in vivo* under UV irradiation of skin are alpha-tocopherol and ubiquinol-10, both in sebum and viable keratinocyte membranes [17, 19, 50–52]. These redox-cycling phenols have been undoubtedly recognised as the first targets of UV oxidation in both three-dimensional skin experimental models [50, 51] or cell-free sets of sebum *ex vivo* irradiation [53, 54]. Only once these borderline lipophilic antioxidant defences of SSL are overcome, there occurs accelerated SQ oxidation; this leaves unchanged the remaining lipid fractions and protects other unsaturated lipids such as cholesterol and, most importantly, the minimal amount of PUFA contributed by pure sebum, namely sebaleic acid (5,8-octadecadienoic acid). This special di-unsaturated fatty acid has been recently proven *in vitro* to be a feasible source of oxidized, biologically active chemoattractant and proinflammatory species, when metabolized by neutrophils and keratinocytes in the skin [55]. *In vivo* instead, the protective effect of SQ on PUFA and the major physiological relevance of SQ peroxides as compared to sebaleic acid oxometabolites were already indirectly proven by the early observations of Morello et al. [56], documenting the absence of significant sebaleic acid depletion in sebum of patients with severe acne, as compared to healthy controls.

Based on the elevated proneness of SQ to photodecomposition, and the many biological effects of UV-induced SQ peroxides it has been recently reproposed that these reactive by-products may be principal physiological molecular mediators of the biological effects of UV irradiation and other pro-oxidants targeting skin [39].

As described above, the protective effects of SQ are counterbalanced by the generation, by the same peroxidative mode, of low-molecular weight and relatively hydrosoluble reactive species [57, 58], chemically derived from the antioxidant action of the triterpene. These by-products are able to diffuse from the skin outer corneum mantel into viable layers and hence target keratinocyte plasma membrane PUFA, thereby causing arachidonic acid decrease and a sequel of consequent biological effects, including the production of a cascade of lipid oxidation reactive nucleophiles, such as 4-hydroxy-2 nonenal (4-HNE). SQ peroxides display the typical bimodal biological behavior of reactive species, exerting *in vitro* a paradoxical effect on keratinocyte cells. At low concentrations and short times of incubation, they stimulate DNA and protein synthesis, whilst inducing cellular damage and inhibiting mitotic activity at higher exposure times and dosages [44]. SQ peroxides also stimulate keratinocytes to release increased amounts of pro-inflammatory cytokines *in vitro* [59] whereas they induce UV-like immunologic effects on the guinea pig ear model* in vivo. *Topical application in concentrations likely produced under physiological UV irradiation of the skin is in fact able to suppress contact hypersensitivity to topically applied haptens like dinitrofluorobenzene (DNFB), as demonstrated by the reduction of lymphocyte infiltrate and the depletion of ATPase positive immunocompetent cells [53].

Among SSL fractions, SQ is the most efficient quencher of singlet oxygen [60], produced under UV irradiation of cutaneous photo-sensitizers or under conditions of phagocyte-driven oxidative burst at skin inflammation sites [39]. SQ peroxide by-products generated by this scavenging action are able to upregulate the release of PGE and cytokines from keratinocytes [59], and through this mechanism they possibly activate melanocyte dendricity and stimulate melanin synthesis in the lower skin layers, as demonstrated on the guinea pig ear model [39]. In this direction, SQ peroxide effects on melanogenesis can in part explain also changes in skin pigmentation *in vivo*.

Taken together, all these results confirm earlier classification of SQ as a sacrificial antioxidant, being extensively degraded under different types of peroxidant stimuli and very prone to photodecomposition [61]. This way, this unique molecule is able to afford protection of human skin surface from further damage to the lipid mantel and to cellular peroxidable targets of the viable layers, induced by the exposure to UV and other sources of ionizing radiation, both natural and iatrogenic. In view of the many ascertained biological effects of UV-induced SQ peroxides, it has been recently re-proposed that these reactive by-products may be principal physiological molecular mediators of the biological effects of UV irradiation and other pro-oxidants targeting skin [39, 62].

The recognition of SQ as a principle target for oxidative stressors on the hydrolipid mantle has been acknowledged in the last decade also for applicative purposes, in that the quantification of SQ oxidative rate, or of SQ hydroperoxide formation, has become a commonly used method to test the protective/antioxidant efficacy of natural or artificial ingredients of formulations directed towards skin protection or sunscreening [48, 57, 61, 63].

**Figure 2:**
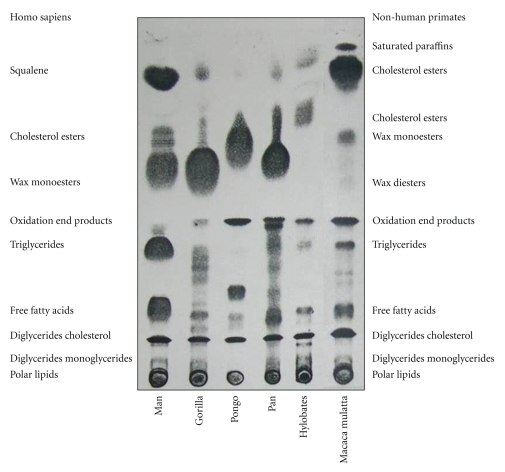
Analysis of skin surface lipid (SSL) fractions of human samples (pooled extracts of 30 healthy individuals) and of non-human primates (from 1 to 5 pooled individuals in each sample) performed by Thin Layer Chromatography (TLC) with double-solvent system resolution. The effective chemical characterization of each lipid fraction was checked by Gas Chromatography—Mass Spectrometry (GC-MS) analysis (*for quantitative results see* Table 1).

In conclusion, though, from the biological point of view, the reason why SQ molecule occurs in high concentration in human, SSL is still considered an enigma. On the basis of all described biochemical properties, it can be sensibly hypothesized that the natural deficiency of squalene oxido-cyclase activity in human sebaceous glands represents an evolutionary advantage, in that SQ is capable of neutralizing reactive oxygen species induced by UV irradiation on the skin, thus behaving as an antioxidant and, indirectly (SQ does not absorb in the UV range), as a natural sunscreen [64]. The skin of monkeys, unlike that of humans, is covered by a large quantity of hair, protecting from UV rays. In the far less hairy human skin, the shield function is reasonably carried out by SQ, in association with the physical defences of stratum corneum and melanin. In contrast to human sebum in fact, SSL of other *hominidae* contains higher levels of cholesterol, and surprisingly no SQ at all. Our group has performed a so far unique study on surface lipid composition in the primate superfamily, proving that SQ is unique to human sebum, and completely missing in the main genera of non-human primates, including those closer to man, the *hominoidea* [65, 66]. Human sebum also contains higher levels of triglycerides and their hydrolysis products and far lower levels of cholesterol. A synthesis of most relevant data is illustrated in Figure 2 and Table 1. In addition, SQ terpene typical of human sebum is also a principal surface lipid of different aquatic mammals, namely otter, beaver, kinkajou, and at least one species of mole [67–70]. In these species, SQ accounts for the essential properties of water repellence and thermal insulation. Other nonaquatic mammals or birds have evolved different cutaneous fats, such as wax esters and wax di-esters, ensuring the same vital properties.

The relevant distance of human sebum in composition and function from the nearest primates, and the close similarity with semiaquatic mammals, bears interesting evolutionary implications and may offer some support to the discussed hypothesis of the origin of man from some semi-aquatic hominids, feeding on fish. Marine food, especially microalgae and seaweeds, as well edible seeds of plants dwelling in Mangrovian habitats, like the genus Amarantus [71], display in fact an unusual rich SQ content. In the light of the so-called Aquatic Ape Theory, that evidences features of convergence among different semi-aquatic species, such as proboscis monkeys, beavers, sea-otters, hippopotamuses, seals, sea lions, and walruses [72], these new biochemical data on human sebum squalene offer indeed new space for speculation. 

## 3. Microbial Impact on SSL Composition and Function

As previously mentioned, the epidermal surface hydrolipid layer can also be viewed as the growing medium for residential saprophytic microbial skin flora. The distribution and density of bacterial and yeast population at cutaneous surface is dependent on host age and on environmental factors such as sebum secretion, occlusion, temperature, and humidity [73, 74]. Sebum produced in the pilo-sebaceous gland is composed purely of squalene, waxes, and triglycerides [34]. Once secreted, this rich medium is immediately colonized by various lipotrophic biologic agents, the development of which is controlled by several defensive humoral mechanisms and by the contact with ambient oxygen. Bacterial/yeast lipase activity is the main responsible of FFA presence on the skin. Oxygen and micro-organisms transform sebaceous moiety and hydrolyse triglycerides to FFA, with consequent relevant alterations of the SSL pattern in cases of pathological microbial colonization of the skin.

This is the case of acne, where a role for *Propionibacterium* infection is claimed among the main aetiopathogenic triggers [75]. Here, FFAs metabolically generated by this lipophilic bacterium account for the chronic inflammatory reaction and the fibrogenetic action on infundibulum epithelium, thus sustaining comedones, pustules, and nodules formation [27]. Similar mechanisms have been claimed to explain at least partially the chronic inflammatory process characterizing atopic (AD) and seborrheic dermatitis (SD) [76, 77]. *Pityrosporum ovale* is a lipophilic saprophyte belonging to the normal skin flora, mainly localizing in the horny layers, and in the upper tract of the sebaceous follicle. In the scalp, for instance, *P. ovale* constitutes up to 46% of the cutaneous flora in the healthy subject, while it may increase up to 82% in SD patients. The evidences of defective cell-mediated response to *P. ovale* and to *Candida albicans* in SD patients [78], and the elevated incidence of SD in HIV^+^ and AIDS patients [79], support the hypothesis that a deficit of cell-mediated immunity may play an aetiological role in the disease. A long-lasting pro-inflammatory action of *P. ovale* is to be taken into account, the yeast being highly immunogenic, able to activate complement and to produce pro-inflammatory reactive oxidized metabolites of selected polyunsaturated SSL, including SQ [34]. Alterations of SSL composition, along with oxidative by-products of SSL irradiation during antitumor PUVA or narrow-band UV-B phototherapy, induce similar immunologic impairment locally on skin. This causes an increased incidence of parasite skin colonization, like, for example, the infection caused by the Demodex mite [80], an intrafollicular parasite feeding on sebum, and also frequently causing blepharitis [34].

Antimicrobials may reduce the density of the resident pathogenic or saprophytic skin flora, but they do not completely eliminate it. While antimicrobials may cause irritant and allergic contact dermatitis, no evidence exists that their use may change the ecology of resident bacteria on the skin, thereby leading to the overgrowth of pathogenic bacteria [74]. As a consequence, antibiotic/antimycotic treatment is not the elective approach to most of these skin diseases. The antimicrobial function of FFA generated on sebum is an essential factor for the homeostasis of skin microbial colonies, and changes in sebum fatty acid composition are a main cause of microbial alterations on the pathological skin [81, 82]. In this respect, skin lipidomics are definitely expected to offer important diagnostic and therapeutic solutions in the near future.

Due to its high levels of unsaturation, SQ in particular has been proposed as the precursor of highly toxic pro-inflammatory mediators, produced by bacterial or yeast lipoperoxidase activity [45]. A further well-characterized example of an aetiologic involvement of SSL in skin pathology is offered by pityriasis versicolor, a pigmentation disease featured by large achromial spots occurring on skin areas with highest concentration of sebum lipids, where *Pityrosporum orbiculare *finds optimal dwelling conditions. This skin saprophyte in some individuals becomes pathogenic, due to still unknown factors. *In vitro* cultures of *P. orbiculare *have documented a markedly increased peroxisomal lipid oxidation activity induced by the same unsaturated fatty acids present on the skin, namely linoleic acid [83], giving rise to hydrogen peroxide through a Fenton mechanism and the subsequent generation of hydroxyl radicals. SQ, which is not a substrate for lipoxygenase and is not metabolized by *Pityrosporum*, may reasonably be peroxidized *in vivo* due to the metabolic activities of yeast peroxisomes. In yeast cultures supplemented with linoleic acid plus SQ, a marked increase in lipoperoxide formation was observed [83], with the generation of the same toxic and unstable oxidation products (trans-trans farnesal and SQ epoxides) formed under experimental high-dosage UV irradiation of sebum and also identified on skin *in vivo* [45].

This equivalence substantiates the role of SQ peroxides in the development of the clinical features of pityriasis, possibly accounting for melanotoxicity and cutaneous depigmentation [84]. In this connection, the early demonstration that vitamin E supplementation suppresses the induction of peroxisomal beta-oxidation and catalase activity induced by linoleic acid offers promising clues for new treatment approaches [85].

## 4. Skin Surface Lipids as Biomarkers of Skin Disease and Aging

All surface lipids examined so far play a role in the complex signaling network originating at the epidermal level, so that skin can no more be viewed as a specialized wrapping material protecting internal organs from environment and guaranteeing the main function of permeability barrier homeostasis, but more extensively as a complex organ actively communicating with the external world [86]. As a consequence, thorough revision has been made of the traditional concept that only mere quantitative alterations of sebum, without any concern for sebum quality and composition, accounted for skin diseases associated with seborrhea or sebostasis. The multifaceted functions of the sebaceous gland are hence gaining momentum [25, 87], as the dysfunction of enzymes synthesizing or metabolizing SSL has been found in different disease states, along with altered sebum antioxidant levels. As widely discussed above, SSLs are subjected to hydrolysis and oxidative processes, giving rise to biologically active by-products, which are critically modulated by local lipid soluble antioxidant levels, actively transported to the skin surface at the level of the pilosebaceous unit. Indeed, SSL composition has been found significantly altered in amount and quality in different skin diseases as well as in the aged skin.

In both atopic and seborrheic dermatitis, we and other authors reported a marked reduction of skin total surface lipids [29, 30]. It was shown that SSL of children and adults with atopic dermatitis present depleted levels of the lipid fractions of sebaceous origin, SQ, and wax esters and correspondingly increased levels of free and esterified cholesterol. Analogous alterations were found in seborrheic dermatitis, in HIV-negative and HIV-positive patients, these latter suffering from SD with increased frequency as compared to healthy population. These alterations of SSL composition, summarized in Table 2, were associated with significant systemic depletion of the main lipophilic antioxidant levels and detoxifying enzymatic activities, mainly vitamin E, ubiquinol-10, and erythrocyte glutathione peroxidase [88, 89].

In the acne pathogenic process, SQ peroxides most likely play a role [90], in that SQ monohydroperoxide has been proven to be highly comedogenic when topically applied on skin in the animal model [91]. Sebum SQ fraction was found increased by 2.2-fold in a group of patients affected with moderate to severe acne, as compared to controls, reaching 20% of the total sebaceous lipids [92]. The SSLs undergo important qualitative and quantitative modifications due to photo-aging, where SQ peroxides seem again to play a major role, mimicking the effects of chronic UV irradiation when applied experimentally on the skin [93, 94]. SSL may hence represent a very efficient marker of the sun-protecting efficacy of chemical sunscreens. Experimental UV irradiation of a series of commercial chemical filters in the presence of SQ in physiological concentrations led to the conclusion that all most common UV filters protect SQ from UVB-driven oxidation at different extents (Benzophenone-3 > Octylsalicylate > Parsol MCX > Parsol5000 > Octyldimethyl p-hydroxybenzoic acid (PABA) > Parsol 1789), depending on filter dose and irradiation energy. Conversely, upon UVA irradiation, filters like Parsol 1789 and 5000 and octyl-dimethyl PABA exerted a marked pro-oxidant effect, enhancing SQ peroxides formation possibly through the action of their self-decomposition by-products induced by UVA, deserving careful attention for safety concerns [64, 95].

The external lipid film hence represents a reliable *in vivo* marker of skin disease, easy to monitor through noninvasive analytical techniques [30, 96]. Being the first target of environmental stressors, SSLs also represent a suitable research tool for *in vitro* screening, to study drug delivery through skin, as well as *in vitro* assessment of the chemical reactions of jewellery, textiles, cosmetics, drugs, industrial chemicals, and particles in direct and prolonged contact with human skin [97]. To provide meaningful results, the composition of artificial SSL should accurately mimic human sweat and sebum, and the conditions of the *in vitro* test system should accurately reflect *in vivo* skin conditions. The wealth of results obtained employing artificial sebum formulations is characterized by the poor physiological value of most models, lacking essential components and presenting unrealistic percent ratios of the single molecules. Variables like sweat composition, pH, temperature, and so forth need careful standardization, in order to guarantee reliability of the *in vitro* test system. The combination of tridimensional skin tissue models with SSL formulations bears promising results for *in vitro* applications, providing both corneum and sebum components to the skin barrier functional model.

## 5. The Influence of Diet and Cosmeceutical Intervention

In view of the physiological relevance of the surface lipid composition for an optimal performance of the skin organ, the modulation of skin surface lipids composition may represent a powerful approach to enhance skin photo-protection, to prevent skin ageing, and to control microbial symbiotic and pathogenic colonization and consequent skin diseases. This goal is attempted traditionally through local application of lipid and/or lipophilic antioxidant-enriched cosmeceutical formulations, with immediate beneficial effects, albeit in most cases not durable after treatment discontinuation.

The possibility to control SSL composition and skin function in general by diet or nutriceutical intervention is far more intriguing and promising a perspective. The administration of skin-tropic lipid-based oral supplements has been claimed to be effective, though available reports are sparse [98, 99]. The analysis of the existing literature brings to the conclusion that nutritional factors provide benefits to skin physiology, but information on the effects of low-to-moderate doses of nutrients consumed in the long term by healthy individuals is lacking, as well as are the data on the direct effects on basal skin properties, including hydration, sebum production, and elasticity, up-to-date scant, and often contradictory. The largest part of information refers to PUFA administration as a treatment for scaly skin, the observation that the formation of PUFA oxidation products on the skin can be suppressed by linoleic acid supplementation through vegetable oil sources [21] demonstrates that skin lipid film can be modulated through the diet more efficiently that through topical application. A low-glycaemic diet regimen also proved effective in the normalization of sebum production and composition in acne patients, confirming the feasibility of the control of disease concurrent factors influencing sebaceous gland physiology through the systemic approach [100].

Concerning antioxidant supplementation, Passi et al. [54] reported no significant alteration of SSL after a daily oral dosage 200 mg of CoQ_10_ for 10 days. Oral supplementation of SQ to mice resulted in a marked dose-dependent upregulation of cellular and nonspecific immune functions [13]. In healthy volunteers, low- or high-dosage (13 or 27 g daily) SQ *per os*, *per* 90 days resulted in significant improvement of antiaging effects on photoaged skin, with facial wrinkle decrease, as confirmed by molecular markers of UV-induced skin damage. Facial erythema decrease and pigmentation increase were observed though high dose oral administration of fat induced some side effects [101].

## 6. Conclusions

Data discussed so far allow us to consider skin surface lipids, and in particular its polyunsaturated component squalene, as a main target for pro-oxidant agents on the skin. SQ peroxides generated locally upon natural or therapeutic UV irradiation are mediators of the immunological response and the melanogenic process in the skin. The SSLs represent suitable markers for the diagnosis of skin disease or aging, and for treatment efficacy monitoring, provided that the results are combined with the analysis of the strictly interconnected systemic biomarkers of oxidative damage and antioxidant defences.

## Figures and Tables

**Table 1:** The skin surface lipid (SSL) composition (%) of *Homosapiens* adults and non-human primates (according to [65, 66]).

Lipid Fraction	*Homo sapiens* (*n* = 30)	*Gorilla gorilla* (*n* = 3)	*Pongo pygmaeus* (*n* = 3)	*Pan troglodytes* (*n* = 4)	*Hylobates sp.* (*n* = 1)	*Macaca sp.* (*n* = 5)
Squalene (SQ)	11.8 ± 0.6	n.d.	n.d.	n.d.	n.d.	n.d.
Cholesterol esters (CE)	1.4 ± 0.2	3.5	4.6	9.8	32.6	40.6
Cholesterol (CH)	1.4 ± 0.1	1.7	1.8	1.8	1.9	3.0
Triglycerides (TG)	34.1 ± 6.5	2.3	0.9	5.1	2.0	4.9
Diglycerides (DG)	1.3 ± 0.2	0.8	0.4	1.0	0.2	0.1
Monoglycerides (MG)	0.4 ± 0.1	0.1	tr	tr	0.2	0.2
Wax mono-esters (WE)	24.5 ± 2.1	37.4	25.0	31.3	2.0	15.2
Wax di-esters (WDE)	n.d.	tr	2.0	tr	1.5	2.3
Free fatty acids (FFA)	24.6 ± 6.1	3.4	2.5	7.4	5.0	8.3
Others (paraffins, etc.)	0.5	50.8	62.8	43.6	54.6	26.4

SSLs were sampled from the forehead and upper chest, with diethyl ether cup extraction method [30]. Results for human skin (30 subjects) are expressed as mean ± S.D.; for primates they are the results of the varying number of samples. *Macaca sp.* sample is a pooled extract of 5 samples, consisting in 1 single individual from each of the following species: *Macaca sp., M. mulatta, M. nemestrina, M. assemensis, *and* M. fascicularis.* tr: traces.

**Table 2:** The skin surface lipid (SSL) composition (%) and total lipid content in adult healthy subjects and in adult patients suffering from seborrheic dermatitis (HIV-negative and HIV-positive) and atopic dermatitis (according to [29, 30]).

Lipid Fraction	Groups			
	Healty subjects (*n* = 30)	Seborrheic dermatitis (*n* = 30)	Seborrheic dermatitis (HIV positive) (*n* = 30)	Atopic dermatitis (*n* = 20)
Squalene (SQ)	12.8 ± 0.6	11.7 ± 0.9*	11.5 ± 1.0*	10.8 ± 1.1*
Cholesterol esters (CE)	1.3 ± 0.2	1.0 ± 0.4	1.8 ± 0.5*	2.4 ± 0.6*
Wax esters (WE)	25.6 ± 3.2	23.9 ± 5.1	23.8 ± 4.5	21.7 ± 1.8*
Triglycerides (TG)	36.1 ± 8.4	40.7 ± 10.3	42.3 ± 10.2	32.6 ± 10.6
Free fatty acids (FFA)	21.6 ± 8.8	18.9 ± 9.6	17.4 ± 10.5	28.8 ± 11.4
Cholesterol (CH)	1.2 ± 0.2	1.7 ± 0.5*	1.7 ± 0.5*	2.4 ± 0.4*
Diglycerides (DG)	1.4 ± 0.2	1.2 ± 0.2	1.5 ± 0.2	1.3 ± 0.2
TOTAL LIPIDS (*μ*g/cm^2^)	195.4 ± 20.6	171.2 ± 29.7*	167.2 ± 30.4*	172.6 ± 17.4*

SSL sampled from the forehead, with diethyl ether cup extraction method [30]. Results are expressed as mean ± S.D.

*Significance level versus healthy subjects at *P* < .05.

## Abbreviations

AD: Atopic dermatitis
DNFB: Dinitrofluorobenzene
FFA: Free fatty acids
4-HNE: 4-hydroxy-2 nonenal
MDA: Malonyl dialdehyde
PABA: Para-hydroxybenzoic acid
PUFA: Polyunsaturated fatty acids
SD: Seborrheic dermatitis
SQ: Squalene
SSL: Skin surface lipids.
